# Concordance between *in vivo* and postmortem measurements of cholinergic denervation in rats using PET with [^18^F]FEOBV and choline acetyltransferase immunochemistry

**DOI:** 10.1186/2191-219X-3-70

**Published:** 2013-10-09

**Authors:** Maxime J Parent, Marilyn Cyr, Antonio Aliaga, Alexey Kostikov, Esther Schirrmacher, Jean-Paul Soucy, Naguib Mechawar, Pedro Rosa-Neto, Marc-Andre Bedard

**Affiliations:** 1 Douglas Mental Health University Institute, McGill University, Montreal, QC H4H 1R3, Canada; 2 Université du Québec à Montreal (UQAM), Montreal, QC H3C 3P8, Canada; 3 Montreal Neurological Institute (MNI), Montreal, QC H3A 2B4, Canada

**Keywords:** Acetylcholine imaging, Vesicular acetylcholine transporter, Nucleus basalis of Meynert, Immunolesion, Animal PET

## Abstract

**Background:**

Fluorine-18 fluoroethoxybenzovesamicol ([^18^F]FEOBV) is a radioligand for the selective imaging of the vesicular acetylcholine transporter with positron emission tomography (PET). The current study demonstrates that pathological cortical cholinergic deafferentation can be quantified *in vivo* with [^18^F]FEOBV PET, yielding analogous results to postmortem histological techniques.

**Methods:**

Fifteen male rats (3 months old) underwent a cerebral infusion of 192 IgG-saporin at the level of the nucleus basalis magnocellularis. They were scanned using [^18^F]FEOBV PET, then sacrificed, and their brain tissues collected for immunostaining and quantification of cholinergic denervation using optical density (OD).

**Results:**

For both PET binding and postmortem OD, the highest losses were found in the cortical areas, with the highest reductions in the orbitofrontal, sensorimotor, and cingulate cortices. In addition, OD quantification in the affected areas accurately predicts [^18^F]FEOBV uptake in the same regions when regressed linearly.

**Conclusions:**

These findings support [^18^F]FEOBV as a reliable imaging agent for eventual use in human neurodegenerative conditions in which cholinergic losses are an important aspect.

## Background

The nucleus basalis of Meynert (NBM), located in the basal forebrain, is the origin of particularly dense cholinergic fibers, projecting to the whole cortical mantle [1]. This basalocortical pathway is known to be involved in alertness and cognitive functions [2]. Moreover, this innervation is severely affected in Alzheimer’s Disease (AD) [3], and its density correlates with symptom severity better than other pathophysiological features such as density of amyloid plaques or neurofibrillary tangles [4], which show a ceiling effect very early as the illness still evolve in severity [5]. In this respect, an efficient *in vivo* method to quantify cholinergic innervations would be an asset to track the changes of the disease, even in later stages.

Brain imaging methods validated for quantitative evaluation of the central cholinergic systems *in vivo* are still scarce. Positron emission tomography (PET) imaging agents have been produced for this purpose. They either target the degradation enzyme acetylcholinesterase (AChE), acetylcholine receptors, or the vesicular acetylcholine transporter (VAChT). While the former two types of markers have also been successfully used to detect alterations in the AD brain [6,7], VAChT as a target offers the additional advantage of being present exclusively on the presynaptic cholinergic neurons [8], allowing for more specificity in imaging measures. This has led to the development of many vesamicol derivative radiomarkers [9] which can be used with PET imaging. Vesamicol binds to the VAChT at the so-called vesamicol receptor site, which is non-competitive with acetylcholine binding [10]. It is therefore not affected by changes in endogenous acetylcholine levels or by medication affecting the concentration of the transmitter. This represents a major advantage over other approaches for the purpose of visualizing cholinergic terminals.

Fluorine-18 fluoroethoxybenzovesamicol ([^18^F]FEOBV) is a vesamicol derivative that has been successfully used in both rodents and primates to estimate brain VAChT distribution [11,12]. Its first human use has been described recently for the purpose of depicting its kinetic profile [13]. Its capacity to detect brain cholinergic depletion has also been shown recently in rats [14], although no postmortem confirmation was provided in this study. We aim here to verify the concordance between the *in vivo* usage of [^18^F]FEOBV with PET and the *postmortem* measurement of cholinergic innervation using immunocytochemistry. We hypothesized that [^18^F]FEOBV PET measures would correlate well with postmortem cholinergic markers, both in terms of localization and magnitude.

## Methods

### Animals

All the procedures described here were performed in accordance with the Canadian Council on Animal Care guidelines and were approved by the research ethic boards of UQAM and McGill University. Fifteen adult male Long-Evans rats (3 months old, 250 to 300 g) were used for this study. All rats were housed under standard conditions in a 12-h/12-h light/darkness cycle, with *ad libitum* access to water and food. Each of them underwent a stereotaxic microsurgery aiming at selectively lesioning the NBM cholinergic neurons. PET imaging with [^18^F]FEOBV was performed 2 weeks later, and animals were sacrificed the same day for *ex vivo* immunocytochemistry.

### NBM immunolesioning

Selective lesions of the NBM cholinergic neurons were performed with a unilateral (left hemisphere) intraparenchymal injection of the immunotoxin 192 IgG-saporin [15]. Rats were first anesthetized using an induction chamber (isoflurane 3% to 5%, oxygen 0.8 to 1.5 L/min) and placed in a stereotaxic frame for rodents, where anesthesia was maintained (isoflurane 2% to 3%, oxygen 0.4 to 0.8 L/min) via a facemask mounted on the upper incisor bar. A dose of 0.2 to 0.25 μg of the immunotoxin 192 IgG-saporin (lot 64–124, Advanced Targeting Systems, San Diego, CA, USA) was infused with a microsyringe in the left hemisphere, at the NBM level. The stereotaxic coordinates for the NBM were the following: 1 mm posterior to the bregma, 2.8 mm lateral to the midline, and 7.6 mm ventral to the cranial surface [16]. No further experimentations were performed on the animals for 2 weeks following surgery to allow full recovery.

### PET acquisition and analyses

On each scanning day, [^18^F]FEOBV was synthesized using a modified method [17] originally described by Mulholland [18]. A *levo* enantiomerically pure precursor (ABX GmbH, Radeberg, Germany) was used, labeled with [^18^F] using a SCINTOMICS (Lindach, Germany) hotbox module, resulting in (−)-[^18^F]FEOBV, which is the only enantiomer showing high affinity for VAChT [11].

All rats were scanned using a CTI Concorde R4 microPET for small animals (CTI, Siemens, Munich, Germany). Each PET session consisted of a 10-min transmission, followed by a 60-min emission scan. PET scans were conducted under light anesthesia (isoflurane 2%, oxygen 0.5 L/min) delivered by a nose cone. Temperature, heart rate, and blood pressure were monitored throughout the procedure using a BIOPAC (Goleta, CA, USA) system. After the animal was placed in the scanner, with the brain positioned at the center of the field of view, the transmission scan was obtained using a rotating [^57^Co] point source. Emission scans were initiated immediately after the transmission scan with a bolus injection of 11.1 to 19.7 MBq (SA = 42.51 to 241.48 TBq/mmol) of [^18^F]FEOBV administered in the tail vein. List mode data was histogrammed into 27 sequential time frames of increasing duration (8 frames × 30 s, 6 frames × 1 min, 5 frames × 2 min, 8 frames × 5 min) over 60 min. Images were reconstructed using a maximum a posteriori algorithm, normalized, and corrected for scatter, dead time, and decay.

Imaging analysis was conducted using minctools (http://www.bic.mni.mcgill.ca/ServicesSoftware). Time-averaged tissue radioactivity images were manually co-registered to a standard rat histological template [19] using seven degrees of freedom (rigid body transformation plus one scaling constant). The image outcome measure distribution volume ratio (DVR) was estimated using a reference tissue-based graphical method for reversible ligands [20]. The cerebellar cortex served as a reference region due to its negligible amounts of cholinergic markers, as revealed by histological studies [21,22]. [^18^F]FEOBV DVR was estimated for every dynamic scan. To estimate cerebral blood flow distribution, relative delivery (R_1_) parametric maps were generated using a simplified reference tissue model [23]. The resulting DVR and R_1_ images were convolved using a Gaussian kernel (FWHM = 1.2 mm).

### Immunocytochemistry and optical density

Following PET acquisition, rats were deeply anesthetized and sacrificed through intraperitoneal injection of ketamine (65 mg/kg), xylazine (13 mg/kg), and acepromazine (1.5 mg/kg) in sterile normal saline and were then transcardially perfused with phosphate-buffered saline (PBS) followed by approximately 300 mL of fixative (4% paraformaldehyde in 0.1 M phosphate buffer). The brains were postfixed for 24 h in this solution and stored in sucrose solution (30% in 0.1 M PBS) and 72 h at 4°C before being cut on a freezing microtome. Coronal 5-μm-thick sections were serially cut from the prefrontal cortex to the cerebellum.

One out of every eight sections was processed for ChAT immunocytochemistry, using a mouse monoclonal antibody raised against whole, purified rat brain ChAT-17. This antibody displays a very high affinity (3 × 1,011 L/M) for ChAT and was used according to a standardized protocol [24]. The free-floating sections were rinsed (3 × 5 min), incubated for 2 h in a blocking solution of PBS containing 2% normal horse serum (NHS; Vector Labs, Peterborough, UK) and 0.2% Triton X-100 (Fisher Scientific, Denver, CO, USA), and incubated overnight at room temperature in the same solution containing 2 μg/mL of monoclonal anti-ChAT antibody for the ChAT-immunostained sections. After being rinsed in PBS (3 × 5 min), sections were incubated for 2 h in biotinylated horse anti-mouse, secondary antibody (cat. #BA-2000, Vector) diluted 1/200 in PBS containing 2% NHS, rinsed in PBS (3 × 5 min), and processed with avitidin-biotin complex procedure (ABC Kit, Vectastain Elite, Vector) for 1 h. The immunoperoxidase labeling was revealed for 3.5 min with a diaminobenzidine kit (Vector). After being rinsed in PBS (3 × 5 min), sections were rinsed in ddH2O for 5 min, transferred to PBS, mounted onto glass slides, air-dried, counterstained with cresyl violet, dehydrated in ethanol, cleared in xylene, and coverslipped with Permount (Fisher Scientific).

The cortical cholinergic denervation was estimated by optical density (OD), obtained from digitized images of the ChAT-immunostained sections. This work was carried out using Image-J (NIH Research Services Branch, Bethesda, MD, USA). OD values ranged on an arbitrary scale from 0 (lowest density) to 3 (highest density). The two brain regions of interest (ROI) used to measure OD were the whole cortical mantle between the prefrontal (+4.20 mm from the bregma) and parietal (−1.44 mm from the bregma) areas, and a subregion of this territory defined as the anterior primary sensorimotor cortex (top half of the dorsolateral convexity, between +4.20 and +0.24 mm from the bregma), where [^18^F]FEOBV DVR was found to be particularly reduced following the immunotoxic lesions. OD values were normalized using values in the corpus callosum, where almost no ChAT-positive reaction products could be identified. Mean OD in each ROI was computed separately for the left and right hemispheres.

### Statistical analyses

In order to quantify lesion-induced [^18^F]FEOBV binding losses and cerebral blood flow changes, a voxel-level analysis was done to compare the DVR and the R_1_ of the 15 lesioned rats to those of 14 previously studied normal rats [14]. After cluster-level multiple comparison correction [25], clusters of adjusted *t* values above 2.05 (*p* < 0.05) were considered significant. In each ROI, the mean OD of the lesioned and non-lesioned hemispheres was compared using a repeated measure *t* test. A linear regression was performed between the mean OD and PET [^18^F]FEOBV DVR at the voxel level, with a threshold of *r* > 0.53 (*p* < 0.05).

## Results and discussion

### Results

**Figure 1:**
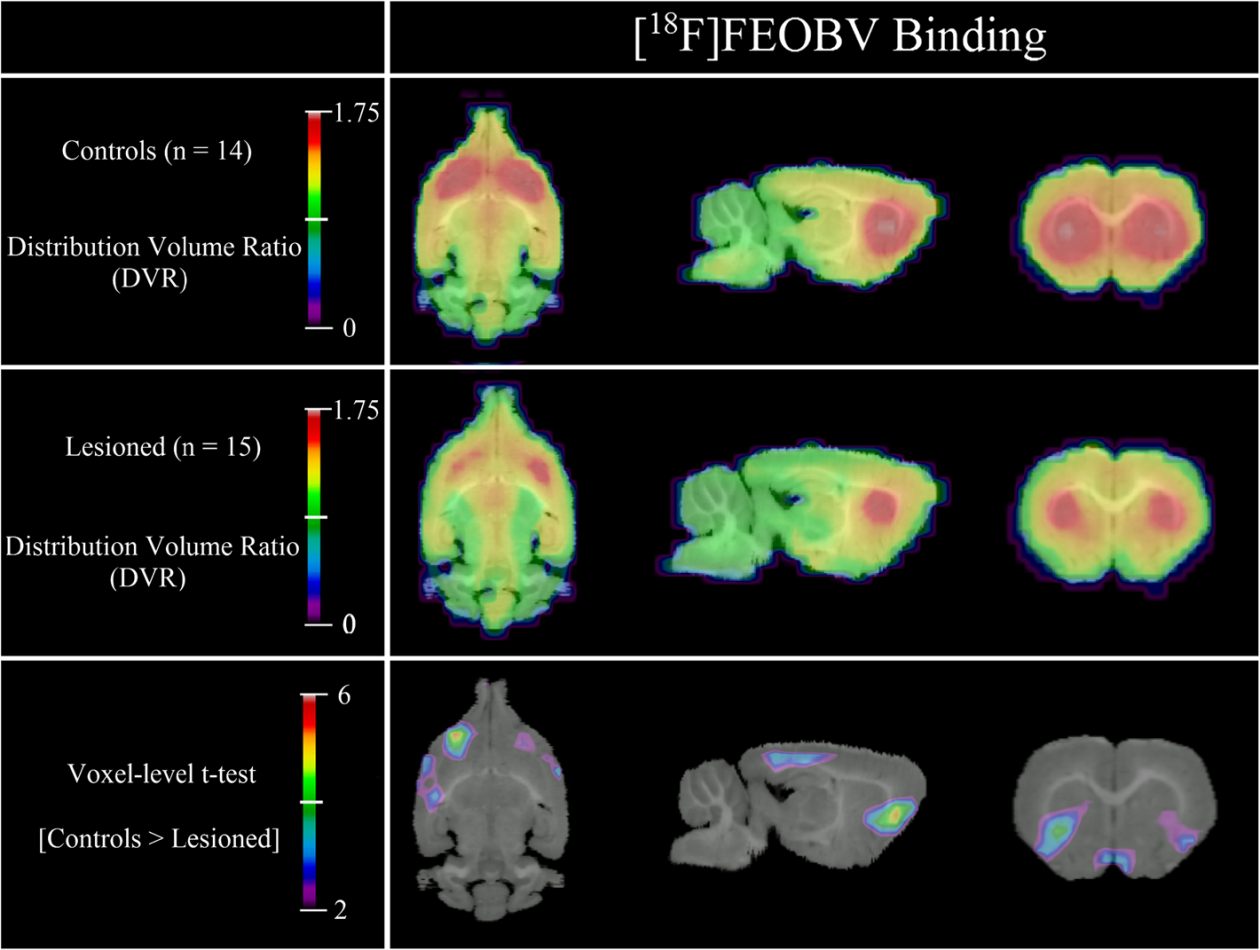
**[**^**18**^**F]FEOBV binding.** Lesioned rats have lower DVR in clusters located in the ventral-frontal cortex of the left (34.56 mm^3^) and right (21.17 mm^3^) hemispheres. Compared to controls, the DVR of the lesioned rats are lower by 22% in the left cluster and 19% in the right one.

As seen in Figure 1, [^18^F]FEOBV DVR of lesioned rats was lower by 22% in comparison with those of normal controls, for a cluster corresponding to the ventral area of the left frontal cortex (34.56 mm^3^, peak *t*(27) = 5.45, *p* = 0.0001). A second, smaller significant cluster can be found in the equivalent area of the right hemisphere (21.17 mm^3^, peak *t*(27) = 3.2, *p* = 0.0004), which corresponds to a 19% difference from normal controls. R_1_ parametric maps of lesioned rats did not differ significantly from those of non-lesioned animals.

**Figure 2:**
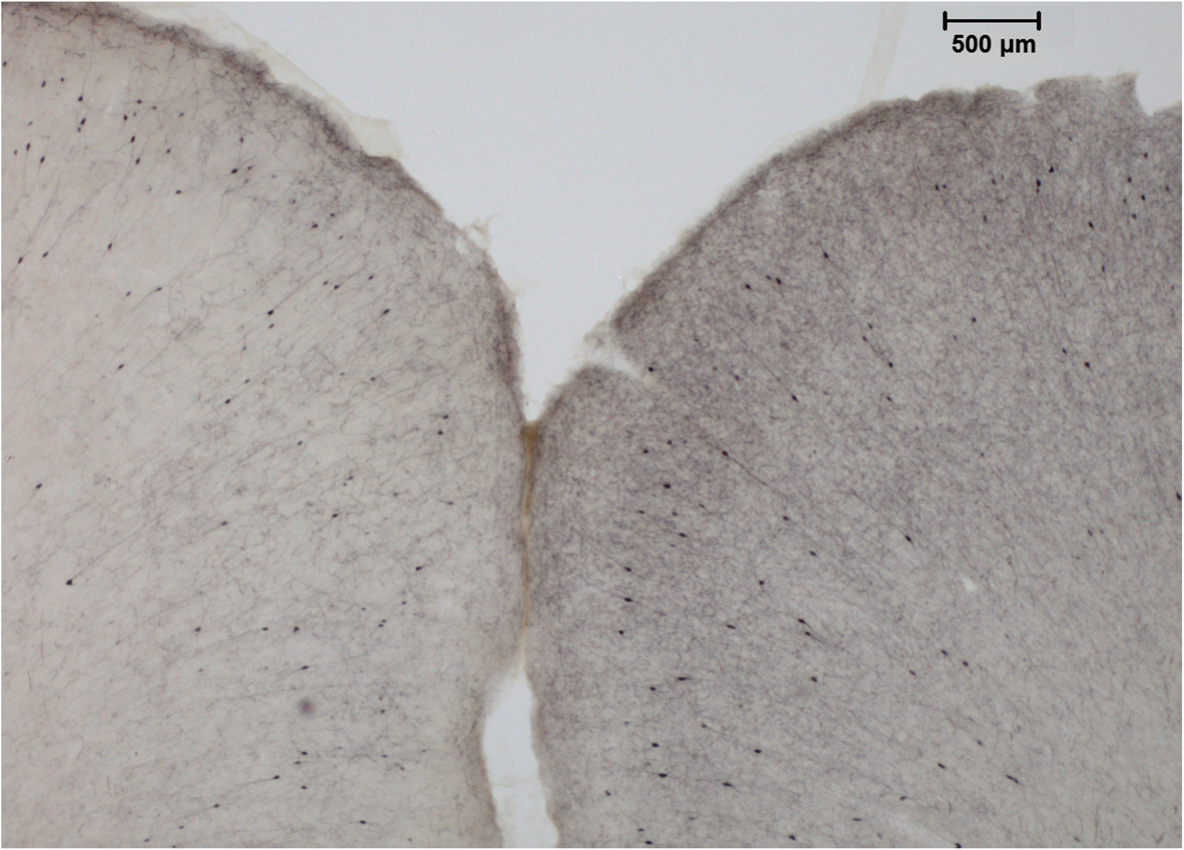
**Anterior cingulate immunocytochemistry.** Example of cortical ChAT immunocytochemistry after unilateral NBM lesion. A very clear difference can be seen between the two hemispheres in the anterior cingulate area (AP = +3.7 mm from the bregma).

**Figure 3:**
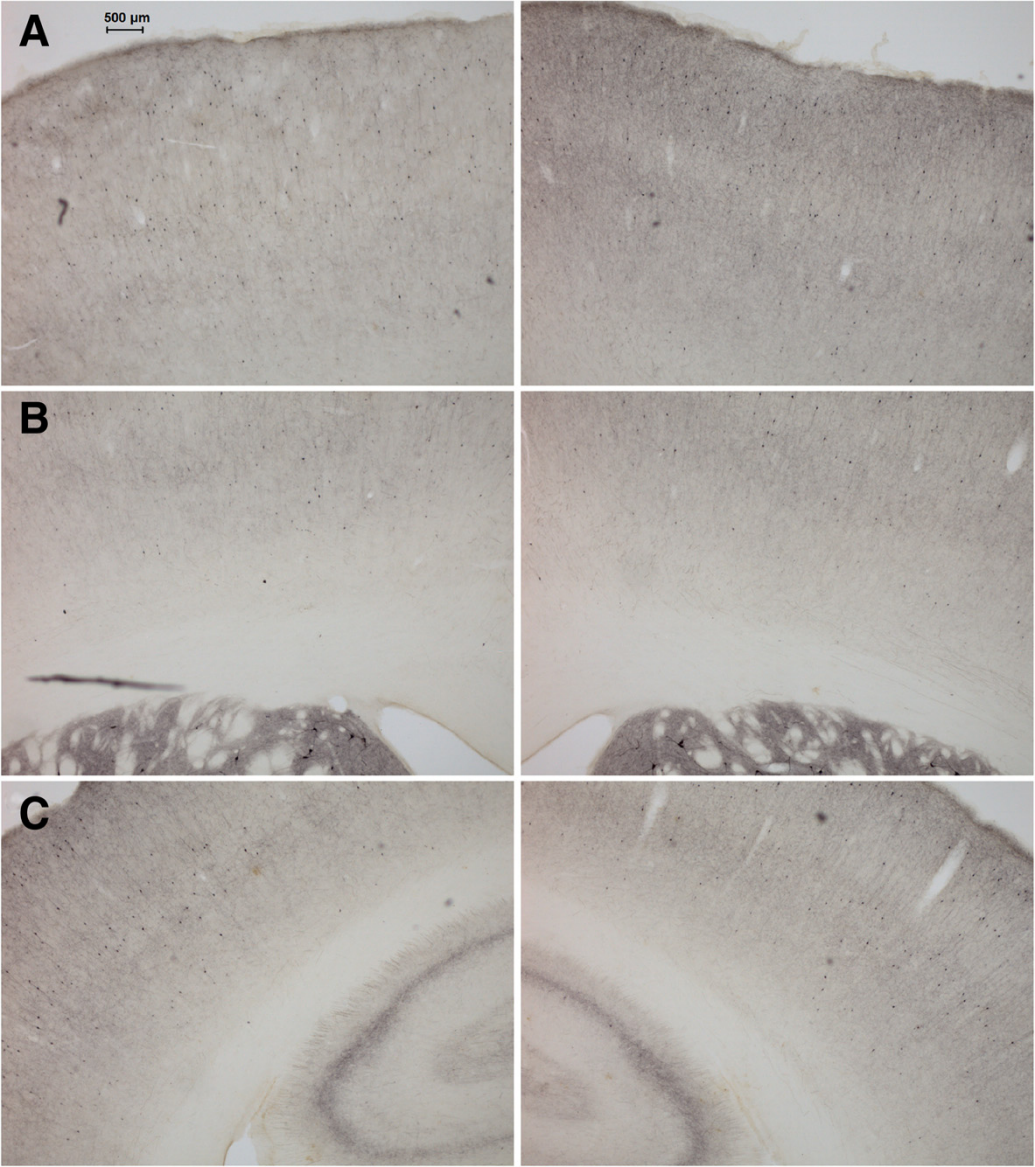
**Anteroposterior immunocytochemistry trend.** Examples of ChAT immunocytochemistry at different anteroposterior locations. The lesioned hemisphere (left column) has a distinct loss of ChAT availability when compared with the control hemisphere (right column). Note the anteroposterior trend: highest interhemispheric differences can be observed in anterior regions such as **(A)** the cingulate and motor cortices (AP = +2.5 mm from the bregma), **(B)** with smaller differences in frontal sensorimotor regions (AP = +0.2 mm from the bregma), and **(C)** no quantifiable effect in the parietal cortex (AP = −2.6 mm from the bregma).

OD analyses revealed higher values in the right hemisphere (non-lesioned) with an average of 17% (*t*(14) = 4.98, *p* = 0.0002). Interhemispheric differences ranged from 0% to 40%, with higher differences being located predominantly in the frontal cortical areas, such as the cingulate, sensorimotor, and orbital cortices. Smaller losses were observed in the insular cortex, while no interhemispheric difference can be detected in the parietal regions (see Figures 2 and 3).

**Figure 4:**
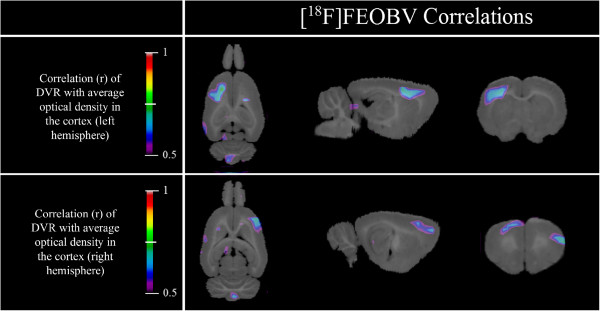
**[**^**18**^**F]FEOBV correlation maps.** Left cortex optical density of ChAT-immunostained slices correlates with [^18^F]FEOBV DVR in a cluster located in the left frontal cortex. Right cortex optical density correlates with a cluster in the right frontal cortex.

**Figure 5:**
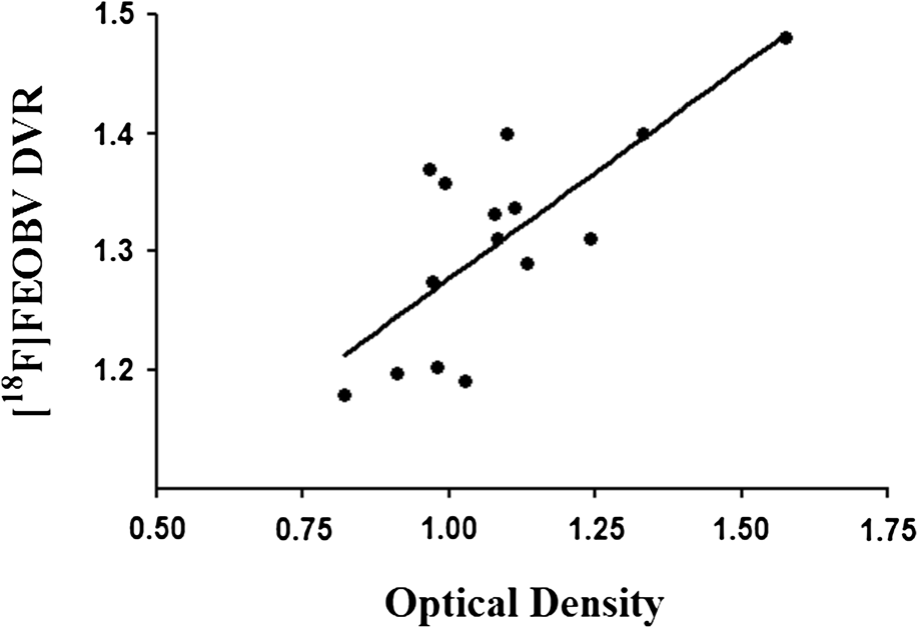
**Localized[**^**18**^**F]FEOBV correlation.** There is a 75% correlation between the average [^18^F]FEOBV DVR in the left cortical cluster and the optical density of the corresponding region in immunohistochemistry.

Regression analysis between the [^18^F]FEOBV DVR at the voxel level (see Figure 4) and the OD values for the whole cortex of the left hemisphere reveals a correlation cluster in the dorsal area of the left frontal cortex (26.35 mm^3^, peak *r*(12) = 81%, *p* = 0.0004). Conversely, OD values of the whole right hemisphere correlate significantly with a symmetrical (although smaller) cluster of [^18^F]FEOBV DVR in the right frontal cortex (17.28mm^3^, peak *r*(12) = 73%, *p* = 0.003). When using only OD values restricted to the same cortical area as the [^18^F]FEOBV DVR cluster in the dorsal left frontal cortex (see Figure 5), a significant correlation of 75% (*p* = 0.002) was still present.

### Discussion

This study aimed to demonstrate the accuracy of [^18^F]FEOBV PET as an *in vivo* measure of cholinergic terminal density. We have shown here that cortical deafferentation resulting from upstream NBM immunolesioning can be estimated by [^18^F]FEOBV PET with DVR measurements, yielding comparable results to those obtained by postmortem quantification. Indeed, the magnitude of the lesions observed with immunocytochemistry and OD in the cingulate, motor, and orbital cortices closely followed what was observed with the [^18^F]FEOBV parametric map. The only exception was the small OD decreases in the endopiriform and insular areas, which were not detected *in vivo*. This may either indicate a lower sensitivity of [^18^F]FEOBV to detect modest changes or a spillover effect from the adjacent caudate-putamen, which is the region with the highest [^18^F]FEOBV retention in the brain. Parietal and more posterior associative cortices appear to have been spared from immunolesioning as neither methods showed interhemispheric difference. This is likely an effect of the injection site, combined with a relatively small dose of 192 IgG-saporin, preventing the spread of damage to the posterior cholinergic efferents.

Although the basal forebrain nuclei contribute to the cholinergic innervation of cortical microvessels in the rat [26], the cholinergic lesions performed here do not appear to have had an appreciable impact on relative tracer delivery. This is consistent with the observation that a targeted unilateral 192 IgG-saporin infusion does not induce interhemispheric differences in blood flow [27]. Indeed, studies showing a relationship with cholinergic basal forebrain lesions and vessel innervation or cortical blood flow have typically used broader, less specific lesioning approaches such as intracerebroventricular 192 IgG-saporin infusion [28] or ibotenic acid [26]. Future studies using [^15^O] water or butanol could serve to quantify the exact impact of such cholinergic lesions on blood flow.

The relative range of ChAT OD value (0.82 to 1.58) observed here was wider than that of [^18^F]FEOBV DVR (1.17 to 1.48). Interestingly, human AD studies also consistently report observed VAChT losses to be proportional, yet of lesser magnitude than ChAT decreases; this discrepancy remains poorly understood [29-31]. In terms of effect size, losses in the lesioned (left) cortical hemisphere are similarly measured with both methods. A 17% interhemispheric difference (*d* = 1.12) was measured with immunochemistry and OD compared to a 22% difference (*d* = 2.61) in [^18^F]FEOBV DVR values. It should be stressed however, that OD is a measure known to suffer from a high level of background noise when compared to other more sophisticated methods of immunochemistry quantification, such as stereology. In addition, the contralateral hemisphere had to be used as a baseline measure, which, as shown with [^18^F]FEOBV imaging, is subject to denervation after unilateral NBM lesioning [14]. As such, it would be expected that a more sensitive method, such as a stereological approach, with tissue acquired from non-lesioned rats as a control measure, would have yielded a larger effect size than that of *in vivo* VAChT imaging.

Regression maps between local OD values and [^18^F]FEOBV binding reveal a strong association in dorsal areas. The same areas are also strongly associated where DVR variance is highest, which explains why no specific group differences were found between control and lesioned rats. In contrast, lesions in the ventral regions have much less variability, which results in clear group effects, but no correlation with OD measurements.

It is also important to note that as a vesamicol derivative, [^18^F]FEOBV binds to VAChT, while ChAT was used as a postmortem biomarker. Beyond the validation of [^18^F]FEOBV PET as a marker for quantifying cholinergic survival, the concordance of the two measures further supports the notion that ChAT and VAChT are regionally co-expressed and highly correlated, both under normal and pathological conditions [32].

## Conclusions

In summary, it has been shown to date that [^18^F]FEOBV has desirable kinetic properties for imaging, selective retention in cholinergic-rich brain areas [11,12]. A first *in vivo* human study has also shown a similar distribution in the brain using reference region approaches, with the highest binding in the striatal nuclei and the lowest in the occipital cortex [13]. [^18^F]FEOBV has also been successfully used to differentiate and quantify cholinergic losses associated with normal aging from those resulting from a pathological process, both in rodent models [14] and in human postmortem tissues from subjects with AD [33]. Here, we add the demonstration that *in vivo* measures of cholinergic innervation density with [^18^F]FEOBV strongly correlate with an accepted postmortem measurement of a closely linked parameter. This constitutes strong evidence that [^18^F]FEOBV is indeed an accurate biomarker of cholinergic axon terminals, with great potential for future clinical uses. In the field of AD research, a specific biomarker of cholinergic synapses could serve as an objective measure of intermediary to late stages disease progression, when clinical symptoms are just beginning to manifest. While radiomarkers of cholinergic receptors have already shown promising results toward this end [6], VAChT tracers such as [^18^F]FEOBV could likely further this goal, thanks to their exclusively presynaptic binding. Other possible applications include several more neurodegenerative disorders such as Parkinson’s disease [34], progressive supranuclear palsy [35], as well as multiple system atrophy [36], in which cholinergic systems are known to be affected.

## Competing interests

The authors declare that they have no competing interests.

## Authors’ contributions

AK and ES operated the cyclotron and synthesized the radioligand [^18^F]FEOBV. AA operated the MicroPET scanner and performed the imaging acquisition. JPS oversaw the nuclear medicine aspects of the project (synthesis and acquisition). PRN contributed to the imaging pretreatment and analysis. MC and NM performed the immunocytochemistry assays and quantification. MJP participated in imaging acquisition and immunocytochemistry preparation, performed all analyses, and drafted the manuscript. MAB oversaw and coordinated the entirety of the project. All authors read and approved the final manuscript.
